# Metabolites from South African Medicinal Plants as Dual-Function Inhibitors of the SARS-CoV-2 Papain-like Protease (PL^pro^)

**DOI:** 10.3390/life16030373

**Published:** 2026-02-25

**Authors:** Mmamudi Anna Makhafola, Clarissa Marcelle Naidoo, Chikwelu Lawrence Obi, Benson Chuks Iweriebor, Oyinlola Oluwunmi Olaokun, Earl Prinsloo, Haruhisa Kikuchi, Muhammad Sulaiman Zubair, Nqobile Monate Mkolo

**Affiliations:** 1 Department of Biology and Environmental Sciences, Sefako Makgatho Health Sciences University, Pretoria 0204, South Africa; clarissa.naidoo@smu.ac.za (C.M.N.); lawrence.obi@smu.ac.za (C.L.O.); benson.iweriebor@smu.ac.za (B.C.I.); oyinlola.olaokun@smu.ac.za (O.O.O.); nqobile.mkolo@smu.ac.za (N.M.M.); 2 Department of Biotechnology, Rhodes University, Makhanda 6140, South Africa; e.prinsloo@ru.ac.za; 3 Faculty of Pharmacy, Keio University, Tokyo 105-8512, Japan; halkiku@keio.jp; 4 Department of Pharmacy, University of Tadulako, Palu 94118, Indonesia; sulaimanzubair@untad.ac.id

**Keywords:** SARS-CoV-2 PL^pro^, *Lippia javanica*, *Acorus calamus*, metabolomics, catechin-7-glucoside, S-adenosyl-methionine, molecular docking, enzyme inhibition

## Abstract

The SARS-CoV-2 papain-like protease (PL^pro^) is an essential viral enzyme that promotes viral polyprotein processing while simultaneously suppressing the host innate immune response, which makes it a primary target for developing antiviral drugs. The present study employs a comprehensive approach integrating untargeted metabolomic profiling, in silico molecular docking and dynamics simulations, Molecular Mechanics Generalized Born Surface Area (MM-GBSA) energetic assessments, and biochemical enzyme assays. This integrated method aims to discover natural PL^pro^ inhibitors from two ethnomedicinal plants, *Lippia javanica* and *Acorus calamus*, which have long been utilized in African traditional medicine to treat respiratory diseases. Comprehensive metabolite profiling using untargeted Ultra-Performance Liquid Chromatography–Tandem Mass Spectrometry (UPLC-MS/MS) and Global Natural Products Social (GNPS) molecular networking revealed flavonoid glucuronides and phenylpropanoid derivatives as the major constituents in both plant species. In situ histochemical staining further offered spatial validation of phenolic- and lignin-associated tissues, supporting the phenolic-dominated molecular families detected by GNPS molecular networking. In silico evaluation of six selected compounds demonstrated spontaneous and thermodynamically favorable binding to PL^pro^, with ΔG_bind values ranging from −5.63 to −6.43 kcal/mol. Catechin-7-glucoside emerged as the lead compound, establishing multiple hydrogen bond networks with Asp164, Gln269, Tyr264, and Asn267, supplemented by hydrophobic engagement with Pro247 and Pro248, and π-π stacking with the blocking loop 2 (BL2 loop). Molecular dynamics simulations confirmed the stability of the protein–ligand complexes. Biochemical enzyme assays confirmed concentration-dependent inhibition of PL^pro^ proteolytic and deubiquitinating activity by both crude plant extracts and isolated bioactive compounds. However, S-adenosyl-methionine showed comparatively high PL^pro^ proteolytic activity (IC_50_ 5.872 µM) compared to catechin-7-glucoside, with an IC_50_ of 7.493 µM, exhibiting efficacy similar to the reference inhibitor GRL0617. Both the extracts of *L. javanica* and *A. calamus* have shown significant inhibitory activity while maintaining cell viability in Human embryonic kidney 293T cell (HEK293T) culture models, indicating a favorable safety profile of the tested concentrations. Based on these results, catechin-based polyphenols and phenylpropanoid derivatives appear as promising lead compounds for the development of PL^pro^ inhibitors. To progress toward therapeutic use, further work is necessary in pharmacokinetics, structural optimization, and antiviral validation in cell models.

## 1. Introduction

The COVID-19 pandemic revealed significant vulnerabilities in global health systems and, therefore, highlighted an urgent need for the development of novel, effective, and affordable antiviral agents. Despite the rapid development of vaccines, challenges such as the distribution, emerging viral variants, and drug toxicity persist, driving the search for new candidates from diverse biological sources [1,2]. The SARS-CoV-2 papain-like protease (PL^pro^) is pointed as one of the key targets. This enzyme plays an essential role in viral replication and also suppresses the host’s immune response via the mechanism of deubiquitination and deISGylation [3,4]. Inhibiting PL^pro^ can therefore block the virus and help in restoring antiviral defenses.

To date, numerous PL^pro^ inhibitors have been documented, acting together with GRL0617 as a representative prototype of a non-covalent inhibitor that can enhance the stability of the blocking loop 2 (BL2 loop) to block substrate accessibility [5,6]. However, synthetic inhibitors such as GRL0617 often display low bioavailability as well as high costs and strong side effects [7]. This has therefore increased interest in natural products as sources of bioactive compounds. Plant derived metabolites, such as flavonoids and terpenoids, have demonstrated promising beneficial effects against a variety of viral targets [8,9,10], including preliminary in silico activity against SARS-CoV-2 proteins [11].

In our previous work, we investigated two African medicinal plants, namely *Lippia javanica* (Burm.f.) Spreng (family Verbenaceae) and *Acorus calamus* L. (family Acoraceae) [12,13,14]. Previous phytochemical studies of *L. javanica* and *A. calamus* have described diverse phenolic metabolites, including flavonoids, phenylpropanoid glycosides such as acteoside-type compounds, lignans, and related derivatives, which exhibit antiviral, and antioxidant, and anti-inflammatory activities [12,13,14]. Using a dual-polarity Ultra-Performance Liquid Chromatography–Tandem Mass Spectrometry (UPLC-MS/MS) approach, we built a detailed metabolomic profile for both plants, identifying compounds with favorable drug-like properties [14]. In the previous study, the comparative analysis of these two botanicals was intentional and hypothesis-driven, based on shared ethnomedicinal importance rather than taxonomic proximity. This study now shifts from chemical discovery to mechanistic validation. We focus on how specific metabolites from our prior findings, such as catechin-7-glucoside, quercetin derivatives, and jasmonate-related oxylipins, interact with SARS-CoV-2 PL^pro^. Molecular docking and dynamics simulations provide atomistic insight into binding interactions and stability [15,16,17]. Critically, starting with empirically identified phytochemicals can reduce the risk of false positives common in purely virtual screens. These computations are followed by in vitro PL^pro^ inhibition assays to confirm bioactivity.

Thus, building directly on our earlier metabolomics work, this study examined certain compounds from the plants *L. javanica* and *A. calamus* for their potential to inhibit the SARS-CoV-2 PL^pro^ enzyme. To map binding interactions, complex stability, and free energy, we employed a series of molecular docking, 200 ns molecular dynamics simulations, and MM-GBSA binding energy computations. These computational predictions were subsequently tested in an in vitro PL^pro^ activity assay. This multilayered approach aims to both validate African phytochemicals as potential scaffolds for the development of COVID-19 treatments and provide a molecular explanation for the historic antiviral use of the selected plants.

## 2. Materials and Methods

### 2.1. Plant Collection and Identification

The procedures for the selection, collection, botanical authentication, and deposition of voucher specimens were performed according to the established methodology of Makhafola et al. [14]. Mature adventitious mid-root segments (2–3 mm diameter) of *A. calamus* L. and fully expanded mature leaves (5–10 cm; 3rd–6th node) of *L. javanica* (Burm.f.) Spreng were collected from Hartbeespoort (25.7236° S, 27.9653° E) in the Northwest Province of South Africa, in austral late summer (February). This collection was authorized under a permit (No: CF6-0234) granted by the Department of Agriculture and Rural Development, Nature Conservation, to Prof. Mkolo Nqobile Monate. Following the collection, taxonomists at the National Herbarium verified the identification of the plants and deposited voucher specimens under the accession numbers NR 904 (*L. javanica*) and NR 905 (*A. calamus*).

### 2.2. Cell Viability Assay Procedure Using xCELLigence Real-Time Cell Analyser

HEK293T cells purchased from Cellonex Separation Scientific SA (Pty) Ltd. (Johannesburg, South Africa) were cultured in Dulbecco’s Modified Eagle’s Medium (DMEM) which was enhanced with 10% fetal bovine serum (FBS) (Thermo Fisher Scientific (Gibco), Waltham, MA, USA) and were utilized for the cell viability assay. Cell viability upon exposure to the selected plant extracts was evaluated using the xCELLigence real-time cell analysis system [Real-Time Cell Analyzer-RTCA (version 1.2.1); ACEA Biosciences Inc., San Diego, CA, USA]. Cells were plated at 1.13 × 10^6^ cells/mL in specialized 96-well E-plates and incubated for 24 h to ensure adherence. The cells were treated with the *L. javanica* and *A. calamus* extracts, subsequently, across a concentration gradient (250, 125, 62.5, 31.25, 15.63, and 7.81 µg/mL). An impedance-based system controlled by RTCA software (version 1.2.1) was used to (constantly) monitor the cell proliferation for 98 h, recording Cell Index values every 15 min. Measurements were similarly taken for HEK293T cells exposed to 0.5% dimethyl sulfoxide (DMSO, 0.5%) as well as for untreated control cells.

### 2.3. Molecular Networking Analysis

A Global Natural Products Social (GNPS) molecular network approach was employed to characterize the chemical families identified via MS/MS analysis in *L. javanica* and *A. calamus* extracts. Using the standardized GNPS Feature-Based Molecular Networking (FBMN) pipeline, the workflow facilitated spectral alignment, grouped structurally similar metabolites, and matched spectra to entries in public databases for annotation.

Compound Discoverer 3.0 was used to process the initial UPLC-MS/MS data (in mzML format) obtained in both ESI^+^ and ESI^−^ modes (Supplementary Material link, Figure S1). This process involved spectrum deconvolution, isotope grouping, retention-time alignment, and feature detection. The result was then sent to the GNPS platform for Feature-Based Molecular Networking (FBMN) analysis. It included MS/MS spectra in mgf files and a CSV table of aligned features with m/z–RT pairs and peak areas. GNPS (https://gnps.ucsd.edu, accessed 10 October 2025) was used to create molecular networks, which were then imported into Cytoscape v3.9.1 for visualization. Different MS/MS properties are represented by nodes in these networks, while spectral similarity (cosine score) is indicated by connecting edges. To contextualize the chemical families identified, clusters containing metabolites listed in Table 1 were evaluated in conjunction with in situ histochemical characterization, alongside complementary computational (in silico docking and molecular dynamics simulations) and experimental (in vitro enzyme inhibition) data.

### 2.4. In Situ Histochemical Characterization

Mature adventitious mid-root segments (23 mm in diameter) of *A. calamus* and fully expanded, mature leaves (5–10 cm in length; 3rd–6th node from the apical tip) of *L. javanica* were freshly collected in late February (austral late summer). Leaf maturity was standardized through controlled pruning (defoliation) to synchronize growth, and uniform size, complete lamina expansion, and absence of senescence, subsequently identifying mature leaves. Root maturity was assessed using morphological indicators, including tissue firmness, uniform diameter, and the absence of apical meristematic features. It was further supported by the known developmental timeline of *A. calamus* under comparable environmental conditions, in which adventitious roots reach complete differentiation approximately 4–6 months after seasonal establishment.

Fresh freehand transverse sections were prepared using sterile razor blades, mounted on glass slides, and subjected to histochemical staining. Stained sections were examined and imaged using a Nikon SMZ745T stereomicroscope equipped with LED illumination (Nikon Eclipse Ci-L, Nikon Corporation, Tokyo, Japan) and NIS-Elements L imaging software version 6.02. Specific classes of biomolecules were localized using the following histochemical stains.

#### 2.4.1. Toluidine Blue O

Sections were immersed for 1 min. Toluidine Blue binds tissue anions, producing metachromatic reactions in which carboxylated polysaccharides stain pink, while lignified cell walls stain blue.

#### 2.4.2. Ferric Trichloride (Phenolic Compounds)

Sections were submerged in ferric trichloride solution for 5 min and examined microscopically. Dark green, bluish-black, or black coloration indicated the presence of phenolic compounds, including tannins.

#### 2.4.3. Phloroglucinol-HCl (Lignin Aldehydes)

To detect lignin, sections were stained with a 1% alcoholic solution of phloroglucinol for 2 min, followed by the careful addition of three drops of concentrated hydrochloric acid. Red or pink coloration of cell walls was taken as evidence of lignin deposition.

### 2.5. Selection of Significance Metabolites

The procedure described by Makhafola and co-workers [14] served as the basis for the workflow for determining the set of bioactive metabolites. An Ultimate 3000 LC system and a Q Exactive mass spectrometer (Thermo Fisher Scientific, Waltham, MA, USA) with an ACQUITY UPLC HSS T3 column (100 mm × 2.1 mm, 1.8 µm), using mobile phases consisting of LC-MS-grade acetonitrile, methanol, and formic acid (Merck, Darmstadt, Germany), were used for the untargeted UPLC-MS/MS study. Candidate metabolites subjected to molecular docking, molecular dynamics simulations, and enzymatic assays were chosen based on statistical significance of VIP > 1.5, False Discovery Rate (FDR)-adjusted *p* < 0.05, fold-change (FC > 2.0), safety profiling, and in silico drug-likeness criteria established in our prior dual-ESI metabolomics study [14]. Metabolites selected for subsequent in silico and in vitro evaluation as potential SARS-CoV-2 inhibitors are presented in Table 1.

### 2.6. In Silico Studies

#### 2.6.1. Library Preparation of Metabolites

Using the data from both ESI^+^ and ESI^−^ modes, a compound library was created from the significant metabolites found in *L. javanica* and *A. calamus* extracts. The Human Metabolome Database (HMDB) and ChemSpider provided structural information (in SDF/MOL2 format) for the top-ranked metabolites. Using AutoDockTools v1.5.7 [18], these structures were created by adding hydrogen atoms, assigning torsional bonds, and computing Gasteiger partial charges. Following optimization, the ligand structures were used for molecular docking against the SARS-CoV-2 PL^pro^ protein.

#### 2.6.2. Protein Preparation (PL^pro^)

**Figure 1:**
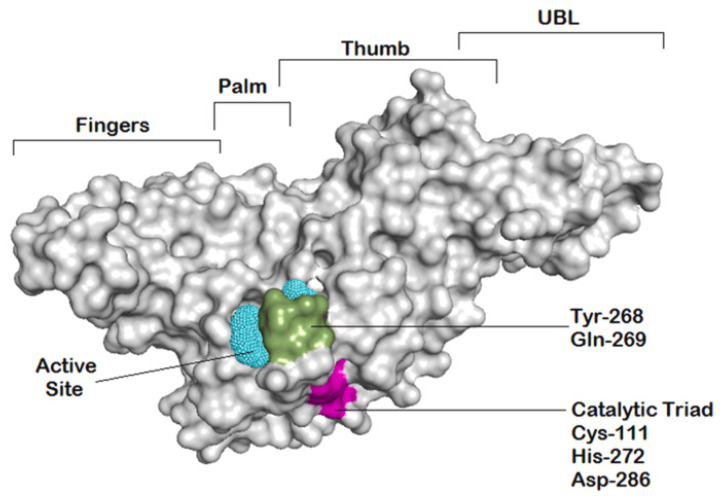
The diagram highlights the essential binding area and the four domains, zinc-finger, thumb, palm, and N-terminal ubiquitin-like domain (UBL) of the SARS-CoV-2 PL^pro^ structure. The cyan mesh shows the potential active site, while the catalytic triad and recognition residues are indicated by magenta and green, respectively [19].

The solid structure of the SARS-CoV-2 papain-like protease (PL^pro^) bound to an inhibitor (PDB ID: 7JRN) [19] was retrieved from the Protein Data Bank. Heteroatoms, water molecules, and the co-crystallized inhibitor were eliminated to obtain the protein ready for docking. After that, Kollman charges were allocated, and polar hydrogens were added. To achieve an energetically stable conformation, the structure was subjected to energy minimisation using the ff19SB force field [6]. The main binding site for docking experiments was determined to be the catalytic triad residues (Cys111, His272, Asp286) (Figure 1).

#### 2.6.3. Molecular Docking

AutoDock Vina v1.2.3 was used to perform docking simulations [4]. A search grid encompassing the full volume of the PL^pro^ active site was positioned over the catalytic triad (Cys111, His272, Asp286). Following the docking of each metabolite into this site, the predicted binding free energy (ΔGbind, kcal/mol) was recorded. For each compound, the conformational pose with the most favorable (lowest) docking score was retained for use in the following molecular dynamics phase.

#### 2.6.4. Molecular Dynamics Simulations

The AMBER22 software version 22 was used to conduct all-atom molecular dynamics simulations [20]. Each docked PL^pro^-ligand complex was solvated in an octahedral box of Transferable Intermolecular Potential 3-Point (TIP3P) water molecules [21], maintaining a minimum 10 Å distance from the protein to the box boundary, and neutralized with sodium or chloride ions as needed. The ff19SB force field [6] was used for protein, while ligand parameters were generated using the general AMBER force field 2 (GAFF2) force field [22]. The simulations were initiated with energy minimization. The systems were then gradually heated from 0 K to 310 K under a canonical ensemble (NVT), followed by equilibration at 310 K and 1 atm in an isothermal–isobaric ensemble (NPT). A production simulation of 200 ns was subsequently performed with periodic boundary conditions and a 2-fs time step. Long-range electrostatics were handled with the particle mesh ewald (PME) method [23], and system coordinates were saved every 10 ps for later trajectory analysis.

#### 2.6.5. Trajectory and Binding Free Energy Analysis

The AMBER trajectory analysis module (CPPTRAJ) module in AMBER22 software was used to do trajectory analysis [17]. The root-mean-square deviation (RMSD) of heavy atoms in each ligand was computed in relation to its initial docked position in order to assess complex stability. The MM-GBSA approach was used to determine binding free energies [24]. One hundred frames were used from each simulation’s equilibrated phase (100–200 ns). The energy contributions from van der Waals interactions, electrostatics, polar solvation, and nonpolar solvation were then added up to determine the ΔG_bind for each complex.

### 2.7. In Vitro Studies

#### 2.7.1. Reagents, Assay Kits and Compounds

SARS-CoV-2 papain-like protease (PL^pro^) activity was evaluated using protease activity kit and deubiquitinase activity kit from BPS Bioscience, San Diego, CA, USA. Both 96-well plate kits supplied consisted of His-tagged PL^pro^, fluorogenic substrates, assay buffer, dithiothreitol (DTT), and the reference inhibitor GRL0617. The top-ranking metabolites from the molecular docking studies, Catechin 7-glucoside (ESI^+^ mode) and S-Adenosyl-methionine (ESI^−^ mode), were procured from Sigma (Darmstadt, Germany) and USP Inc. (Rockville, MD, USA), respectively. All reagents and compounds were stored and handled as per the suppliers’ protocols to preserve stability.

#### 2.7.2. Preparation of Assay Buffer and Enzyme

The assay procedure involved freshly preparing a 1 mM DTT working solution by diluting a 0.5 M stock in the provided assay buffer. To maintain its activity, the recombinant PL^pro^ enzyme was frozen on ice, briefly centrifuged, and separated into single-use aliquots. Deubiquitinase assays required a higher enzyme concentration of 0.7–1 ng/µL (21–30 ng per well), whereas protease assays employed a working dosage of 0.3–0.5 ng/µL (9–15 ng per well).

#### 2.7.3. Sample Preparation

The methanolic plant extracts and test compounds were initially dissolved in DMSO, ensuring a final concentration in all assay wells did not exceed 0.1% (*v*/*v*). The known inhibitor GRL0617 served as positive control. Primary stock solutions were made at 100 times the intended test concentration. These were then diluted 20-fold in assay buffer containing 1 mM DTT to create intermediate working solutions. Testing spans a concentration range of 0.3–100 µM for compounds and 1–300 µg/mL for crude extracts. To measure the background signal, control wells containing only assay buffer (without enzyme) were included on every plate.

#### 2.7.4. Protease Activity Assay

For each assay, triplicate wells of a 96-well plate were prepared with 30 µL of the diluted PL^pro^ enzyme. Control wells received 30 µL of assay buffer without enzyme. A 10 µL volume of either the test compound or the reference inhibitor GRL0617 was then added to the appropriate wells. Following a 30 min pre-incubation at 37 °C, the reaction was started by adding 10 µL of fluorogenic substrate to reach a final concentration of 25 µM. After sealing the plate, it was incubated for 45 to 60 min at 37 °C. A UV/Vis spectrophotometer microplate reader (Turner BioSystems, Sunnyvale, CA, USA) was used to measure fluorescence after incubation, using excitation and emission wavelengths of 360 nm and 460 nm, respectively.

#### 2.7.5. Deubiquitinase Activity Assay

The deubiquitinase assay was conducted following a procedure similar to that of the protease activity test, with adjustments. After dispensing the PL^pro^ enzyme and test compounds as previously outlined, reactions were started by adding 10 µL of a diluted ubiquitinated fluorogenic substrate to reach a final concentration of 250 nM. The plates were also incubated at 37 °C for 45–60 min, and fluorescence was read at excitation 360/emission 460 nm. For analysis, the fluorescence values from blank wells were subtracted from all sample readings.

### 2.8. Data Analysis

All biological tests were performed in triplicate, with data presented as mean ± standard deviation (SD). Dose–response curves were created through nonlinear regression analysis ([inhibitor] vs. response, three parameters) in GraphPad Prism 10 (GraphPad Software, La Jolla, CA, USA). The half-maximal inhibitory concentration (IC_50_) for each compound was derived from the fitted sigmoidal curve, and model fit was verified using the coefficient of determination (R^2^) and residual plot examination. Statistical analyses were performed using one-way ANOVA followed by Tukey’s post hoc test, with comparisons made against the reference inhibitor GRL0617. A *p*-value of <0.05 was considered statistically significant unless stated otherwise.

## 3. Results

### 3.1. Cell Viability Assay Using xCELLigence Real-Time Cell Analyser

**Figure 2:**
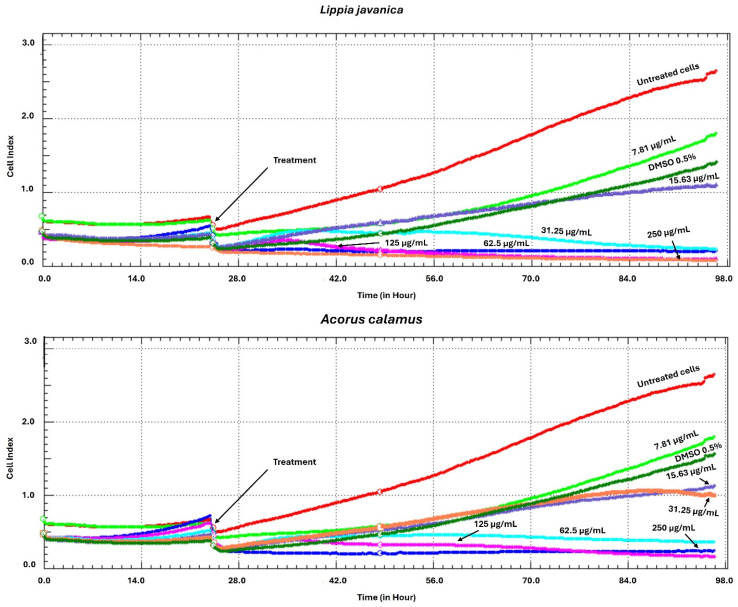
Real-time analysis of HEK293T cell responses following exposure to *L. javanica* and *A. calamus* extracts at concentrations ranging from 250 to 7.81 µg/mL, monitored using the xCELLigence RTCA system.

The xCELLigence Real-Time Cell Analyser (RTCA) was used to evaluate the possible cytotoxicity of extracts from *L. javanica* and *A. calamus* on HEK293T cells. The findings demonstrated that at the lowest concentrations of 31.25, 15.63, and 7.81 µg/mL, methanolic extracts from both plants showed no significant cytotoxicity. According to RTCA findings, cells treated with these lower doses continued to proliferate steadily for 24 to 98 h after treatment. Additionally, extract-treated cells showed an increase in cell index (CI) values that was similar to those of both untreated cells and cells treated (Figure 2).

### 3.2. Molecular Networking Analysis

**Figure 3:**
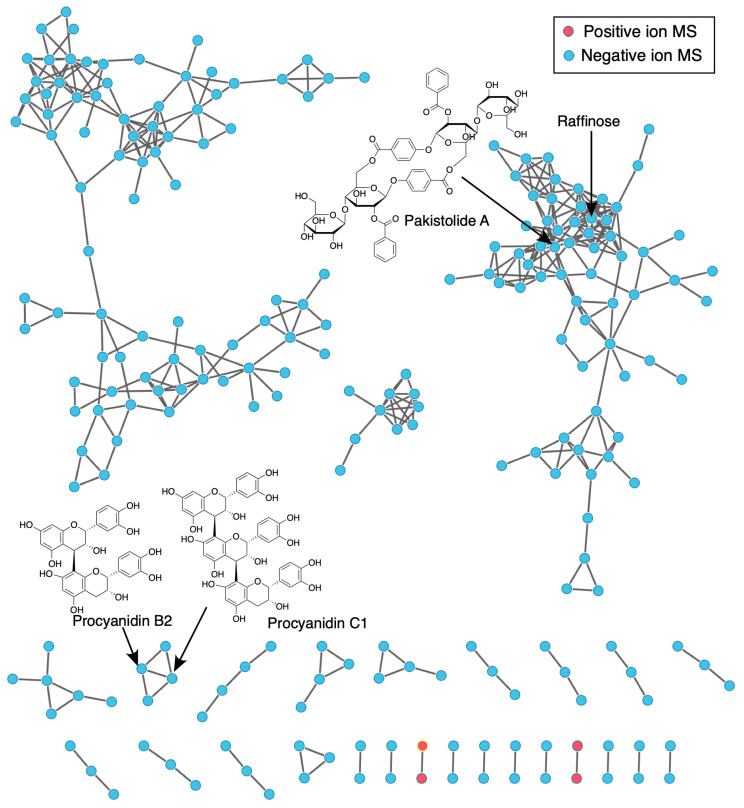
The main chemical families found in *A. calamus* extracts, visualized using the GNPS molecular networks, which are constructed from combined ESI^+^ and ESI^−^ UPLC-MS/MS data.

**Figure 4:**
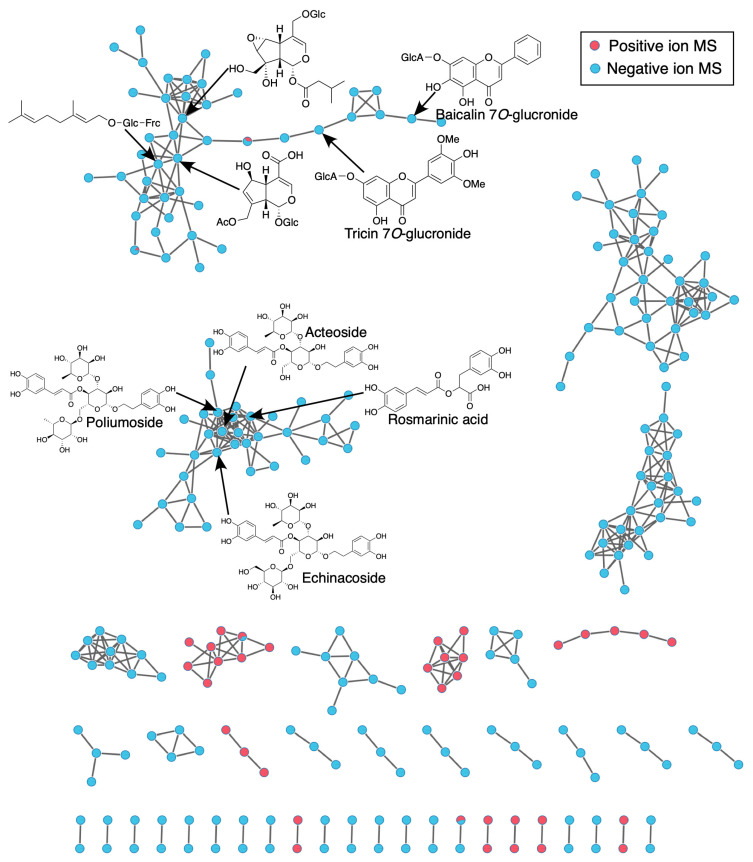
The GNPS molecular networks constructed from positive- and negative-ion UPLC–MS/MS data highlight major molecular families present in *L. javanica*.

Chemical profiling of *L. javanica* and *A. calamus* extracts through molecular networking demonstrated plant-specific clustering patterns linked to their major phytochemical constituents. Condensed tannin clusters containing procyanidin derivatives such as Procyanidin B2 and Procyanidin C1, together with a sesquiterpenoid cluster containing Pakistolide A, were the main characteristics of the *A. calamus* network. Along with a number of unidentified high-molecular-weight clusters, a small carbohydrate cluster containing Raffinose was also found (Figure 3). The *L. javanica* network, on the other hand, exhibited more phytochemical diversity, with notable clusters made up of rosmarinic acid, phenylpropanoid glycosides (acteoside, echinacoside, poliumoside), and flavonoid glucuronides (tricin-7-O-glucuronide and baicalin-7-O-glucuronide). Together with additional unidentified clusters, these metabolites formed dense, flavonoid-rich molecular families (Figure 4).

### 3.3. In Situ Histochemical Characterization

**Figure 5:**
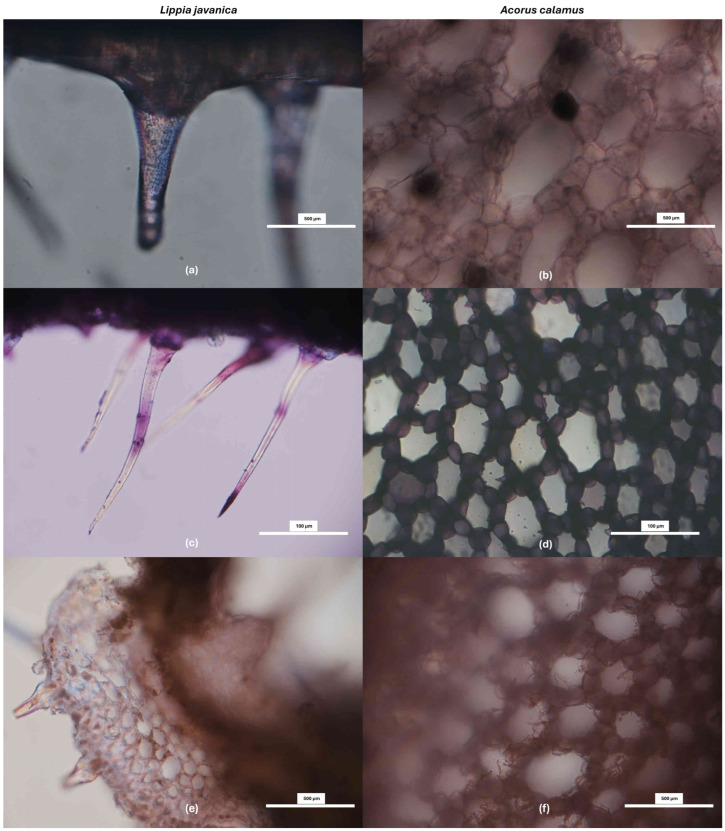
Histochemical staining of *L. javanica* leaves (**a**,**c**,**e**) and *A. calamus* roots (**b**,**d**,**f**) show localization of phenolic compounds (ferric chloride), metachromatic tissue differentiation (Toluidine Blue O), and lignin deposition (phloroglucinol-HCl). Scale bars as shown.

Ferric chloride staining revealed strong phenolic localization in both *L. javanica* leaves and *A. calamus* roots. In *L. javanica*, intense dark bluish-black staining was observed in leaf tissues, particularly in epidermal and associated structures, consistent with the high abundance of flavonoid and phenylpropanoid derivatives detected by GNPS molecular networking (Figure 5a). Similarly, *A. calamus* root sections exhibited pronounced ferric chloride-positive deposits within parenchymatous tissues (Figure 5b), supporting the accumulation of phenolic and lignan-related metabolites identified through untargeted LC-MS/MS analysis (Table 1).

Toluidine Blue O staining revealed distinct metachromatic patterns in both *L. javanica* leaves and *A. calamus* roots, with blue-stained lignified tissues and pink-purple polysaccharide-rich regions, indicating active phenylpropanoid-associated structural differentiation (Figure 5c,d). Consistently, phloroglucinol-HCl staining demonstrated pronounced lignin aldehyde deposition in the vascular and supporting tissues of both species (Figure 5e,f). These histochemical patterns corroborate GNPS molecular networking results (Figure 3 and Figure 4), which identified phenylpropanoid- and lignan-related molecular families as prominent components of the metabolomic profiles.

### 3.4. In Silico Studies

#### 3.4.1. Molecular Docking

Molecular docking results showed that each of the six selected metabolites each formed stable, favorable complexes and interactions with the SARS-CoV-2 PL^pro^ active site, possessing predicted binding free energies (ΔG_bind) between −5.63 and −6.43 kcal/mol (Table 1).

**Figure 6:**
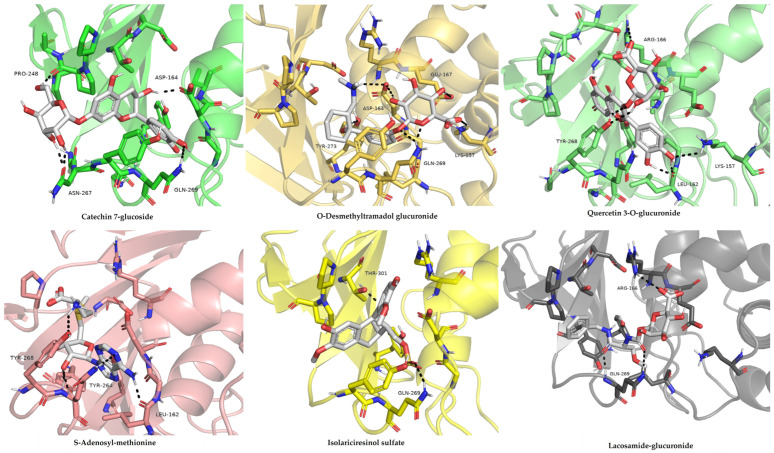
Molecular docking simulations of the six metabolites within the SARS-CoV-2 papain-like protease (PL^pro^) catalytic pocket.

Catechin-7-glucoside demonstrated the strongest predicted binding affinity (−6.43 kcal/mol) (Figure 6). Its favorable docking pose was stabilized by a network of hydrogen bonds with residues such as Asp164, Gln269, Asn267, and Tyr264, as well as backbone atoms of Pro248, Gly163, and Leu162. Hydrophobic contacts with Pro247, Pro248, Tyr268, and Tyr264 positioned the compound’s aromatic ring parallel to the BL2 loop (residues 265, 275). This interaction profile locates the ligand within the substrate-binding cleft near the catalytic triad (Cys111, His272, Asp286). The stability of this hydrogen-bond network in molecular dynamics simulations indicates high shape complementarity and supports a potential non-covalent inhibition mode.

A comparable binding affinity (−5.70 kcal/mol) was demonstrated by quercetin-3-O-glucuronide, which stabilized its position through five hydrogen bonds with Tyr268, Gln269, Asn267, and Asp302 in addition to a polar contact with Gly163 close to the catalytic site (Figure 6). Electrostatic stabilization was enhanced by the glucuronide moiety’s interactions with Lys157 and Arg166. Additionally, quercetin formed π–π stacking interactions with Tyr264, positioning its rings parallel to the BL2 loop. This likely limits the loop’s flexibility, a known mode of action for PL^pro^ inhibitors. Similarly, S-adenosyl-methionine bound (−5.75 kcal/mol) favorably via a hydrogen-bonding network involving Lys157, Asp164, Gly163, and Ser170. The adenosine ring exhibited π–H stacking interactions near Tyr268, while the sulfonium group formed stabilization through electrostatic interactions with Asp302.

O-desmethyltramadol glucuronide exhibited a binding free energy of −5.63 kcal/mol, mostly driven by hydrophobic interactions with Pro247 and hydrogen bonding interactions with Gly163, Tyr268, and Gln269 (Figure 6). Its position was oriented toward the upper part of the binding cleft, partially occupying the substrate-recognition site without approaching the catalytic Cys111 closely. Isolariciresinol sulfate demonstrated a strong polar affinity (−5.66 kcal/mol), which formed hydrogen bonds with Asp164, Tyr264, and Gln269. Its sulfate group engaged in ionic interactions with Lys157 and Arg166, while hydrophobic contacts with Pro248 and Tyr268 anchored the phenylpropanoid scaffold in the pocket. Lacosamide-glucuronide displayed moderate binding (−5.71 kcal/mol), stabilized by hydrogen bonds with Asp164, Gly163, and Tyr268, along with π–alkyl contacts involving Leu162 and Pro247 (Figure 6). The glucuronide group was oriented toward the solvent-exposed region, which likely improved aqueous solubility but limited deeper hydrophobic insertion into the active site.

#### 3.4.2. Molecular Dynamics Simulations

**Figure 7:**
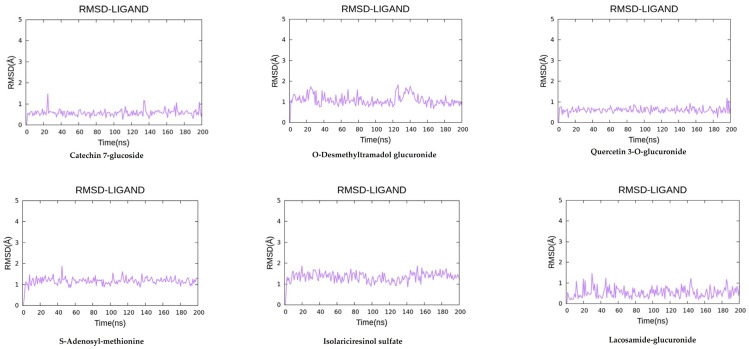
Root-mean-square deviation (RMSD) plots of the ligand backbone atoms during 200 ns molecular dynamics simulations for the top six prioritized metabolites bound to SARS-CoV-2 papain-like protease (PL^pro^).

To authenticate the docking poses and examine the active stability of the PL^pro^-ligand interactions, 200 ns all-atom molecular dynamics simulations were performed on the five highest-ranked compounds (Figure 7). The root-mean-square deviation (RMSD) of the protein Cα atoms demonstrated that each complex stabilized after around 25 ns and maintained this equilibrium throughout the simulation. Catechin-7-glucoside demonstrated the lowest RMSD range (1.5–2.0 Å), suggesting the highest structural stability. Quercetin-3-O-glucuronide (1.8–2.3 Å) and S-adenosyl-methionine (2.1–2.7 Å) exhibited comparable constant patterns. O-desmethyltramadol glucuronide displayed moderate ligand mobility with early stability in the simulation and fluctuating within a narrow RMSD range of around ~0.8–1.5 Å, suggestive of sustained but slightly more flexible interactions relative to flavonoid glucuronides. Isolariciresinol sulfate demonstrated comparable stability with RMSD values showing consistency around ~1.0–1.6 Å with no indication of progressive drift. Moreover, lacosamide-glucuronide displayed the greatest ligand flexibility amongst the tested compounds, reaching RMSD fluctuations of ~1.5–2.0 Å.

### 3.5. In Vitro Studies

#### 3.5.1. Protease Activity Assay

**Figure 8:**
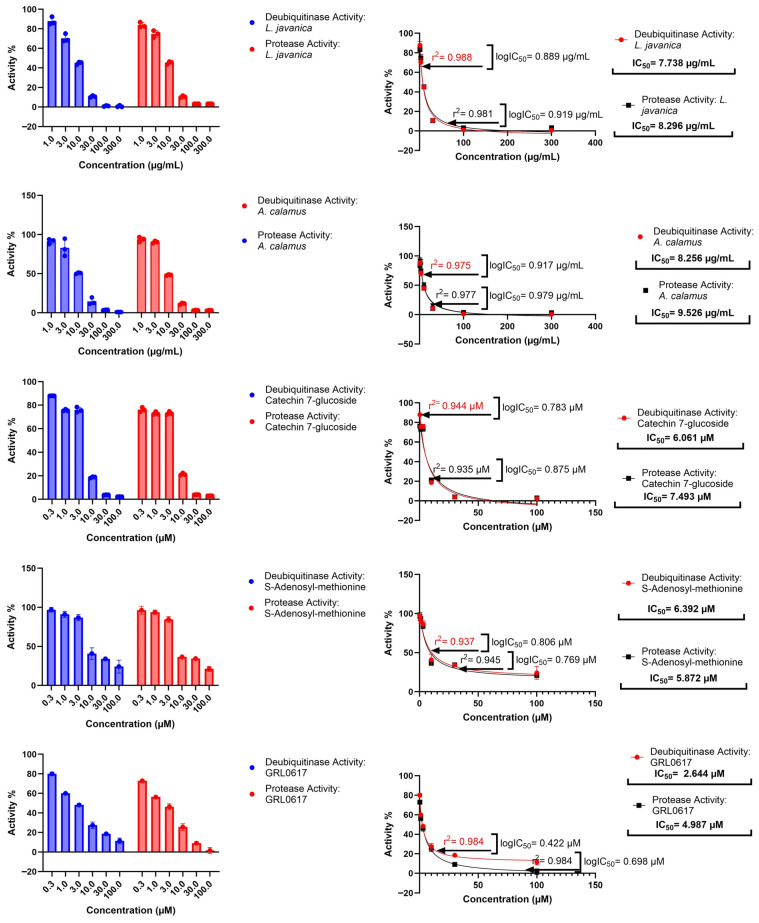
Dose–Response curves showing the inhibition of SARS-CoV-2 PL^pro^ protease and deubiquitinase activity by *L. javanica* extract, *A. calamus* extract, catechin-7-glucoside, S-adenosyl-methionine, and GRL0617. Data represent mean ± SD (*n* = 3); IC_50_ values were derived from nonlinear regression.

All evaluated samples comprising *L. javanica* and *A. calamus* extracts, catechin-7-glucoside, S-adenosyl-methionine, and the control inhibitor GRL0617 inhibited SARS-CoV-2 PL^pro^ protease activity in a dose-dependent manner (Figure 8). While DMSO 0.1% (*v*/*v*) did not inhibit SARS-CoV-2 PL^pro^ protease. Amongst the natural compounds, S-adenosyl-methionine showed comparatively high activity (IC_50_ 5.872 µM) compared to catechin-7-glucoside, with an IC_50_ of 7.493 µM. However, the reference inhibitor GRL0617 had the highest inhibition with an IC_50_ of 4.987, better than the natural compounds. While the *L. javanica* extract had an IC_50_ of 8.296 µg/mL and *A. calamus* extract (with) an IC_50_ of 9.526 µg/mL. At low concentrations (0.3–3 µM or µg/mL) for both natural compounds and plant extracts, protease activity remained high. Intermediate concentration (10–30 µM or µg/mL) reduced activity, while the highest concentrations tested (100 µM or 300 µg/mL) further suppressed the activity (Figure 8).

#### 3.5.2. Deubiquitinase Activity Assay

All the tested samples exhibited concentration-dependent inhibition of PL^pro^ in the deubiquitinase assay, but with greater IC_50_ values than in the protease assay (Figure 8). However, DMSO 0.1% (*v*/*v*) did not exhibit inhibition. The most efficient natural chemical was catechin-7-glucoside (IC_50_ 6.061 µM), while S-adenosyl-methionine displayed an IC_50_ of 6.392 µM. The reference inhibitor GRL0617 with an IC_50_ of 2.644 µM, continued to be a stronger inhibitor than the natural compounds. The extract from *L. javanica* yielded a higher inhibition with an IC_50_ of 7.738 µg/mL compared to the extract of *A. calamus* with an IC_50_ of 8.256 µg/mL. Similar to the protease data, residual deubiquitinase activity was highest at low doses, decreased at intermediate levels, and lowest at maximum values (Figure 8). 

#### 3.5.3. Cross-Assay Comparative Interpretation

**Figure 9:**
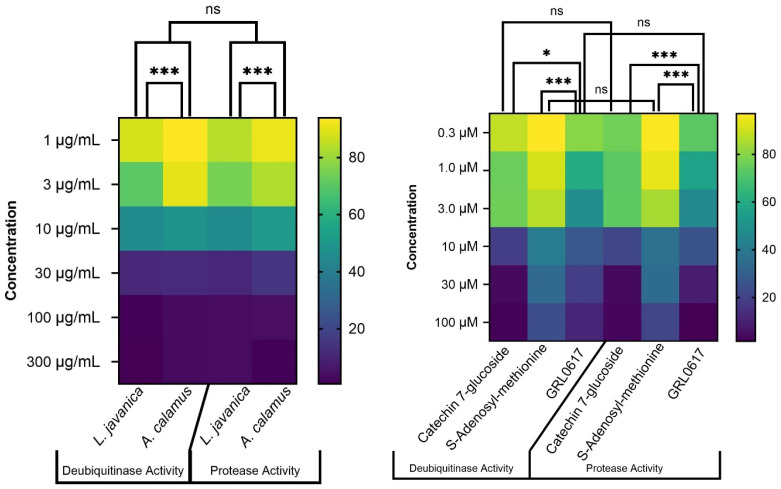
Comparisons of protease and deubiquitinase percent activity for all tested samples. Symbols represent statistical comparisons relative to GRL0617. Key: ns = not significant *p* > 0.05; * = *p* < 0.05; *** = *p* < 0.001.

Comparative analysis of the protease and deubiquitinase assays indicated a uniform order of inhibitory strength for all tested samples (Figure 9). Only catechin-7-glucoside demonstrated protease activity approaching the level of the reference inhibitor GRL0617, at mid to high concentrations, highlighting its promise as a potent natural PL^pro^ inhibitor. Statistical evaluation across both assay types confirmed that the difference in activity between catechin-7-glucoside and GRL0617, although relatively small was significant (*p* < 0.005). In addition, both compounds were significantly more effective than S-adenosyl-methionine (*p* < 0.001). The inhibitory profiles of *L. javanica* and *A. calamus* extracts were similar, with no statistically significant difference between them (*p* > 0.05), especially at the highest concentrations (10–300 μg/mL).

## 4. Discussion

This research provides a multi-layered characterization of *L. javanica* and *A. calamus* as sources of plant-based natural inhibitors of SARS-CoV-2 papain-like protease (PL^pro^) through the integration of metabolomics-guided prioritization, GNPS molecular networking, molecular docking, molecular dynamics simulations, MM-GBSA energetics, and dual enzymatic validation. Consistent data derived from metabolomic prioritization, computational modeling, and in vitro inhibition provide robust conclusions that phenylpropanoid and catechin-type metabolites engage in a mechanistically significant manner with PL^pro^. These metabolites belong to a vast array of phytochemical scaffolds (that are) well represented in the African medicinal flora. It is important to note that the strongest natural inhibitor was catechin-7-glucoside, while S-adenosyl-methionine exhibited moderate activity. This marks a crucial discovery, as low metabolite yield or inconsistent phytochemical fingerprints usually hinder the development of (most) natural antiviral products. The utilization of GNPS molecular networking, in this instance, validated that the identified bioactive constituents belong to highly abundant chemical clusters characteristic of both *L. javanica* and *A. calamus.* Notably, metabolite abundance is not necessarily indicative of biological activity [24]. These findings corroborate the biological validity and facilitate the development of reproducible, standardized botanical extracts for future clinical or pharmacological application [25,26,27,28,29].

The inhibitory mechanism of these compounds against PL^pro^ is supported by their structural alignment to established binding patterns of inhibition. In silico docking simulations indicated that all six selected metabolites occupied the PL^pro^ active binding site through spontaneous, energetically feasible complexes, yielding binding affinities ranging from −5.63 to −6.43 kcal·mol^−1^. Catechin-7-glucoside demonstrated the highest binding efficacy, which was stabilized by an intricate network of hydrogen bonds to Asp164, Gln269, Asn267, and Tyr264, accompanied by interactions of (a) hydrophobic nature with Pro247, Pro248, and Tyr268. The alignment of the compound’s aromatic scaffold was positioned parallel to the flexible BL2 loop (residues 265–275). This region is pivotal for the recognition of substrate in PL^pro^ as key residues such as Tyr268 and Gln269 are known to undergo functional conformational rearrangement during inhibitor binding [28]. The modality of binding aligns in close relation to the inhibitory mechanism of the reference ligand GRL0617. PL^pro^ is inhibited by GRL0617 through the locking action of the BL2 loop in closed conformation, thereby physically occluding the binding cleft and preventing the viral polyprotein’s C-terminal LXGG recognition sequence from reaching the Cys111–His272–Asp286 catalytic triad [29]. This observation is supported by structurally inclined evidence suggesting that compounds engaging with Tyr268 and the adjacent residues significantly restrict the conformational flexibility of the BL2 loop. Subsequently, structural restrictions diminish the ability of the loop to transition to its ‘open’ state, thereby exerting a dual inhibitory effect on the protease and deubiquitinase functional activities of the enzyme [11,29]. Similarly, the binding profile of quercetin-3-O-glucuronide interacted with Tyr268, Gln269, Asn267, and Asp302 through hydrogen bonding, yielding a predicted binding affinity of −5.70 kcal·mol^−1^. Its glucuronide moiety added enhanced structural stability through the formation of electrostatic interactions with Lys157 and Arg166. The flavonoid scaffold of the compound engaged in π–π stacking interaction with Tyr264, and its overall engagement with the BL2 loop aligns with known existing patterns of inhibition for glycosylated flavonoids, which are recognized for their ability to occupy the S3/S4 pocket and effectively obstruct the mobility of the BL2 loop [29]. This framework is further substantiated by comparable binding energies of a favorable nature detected for lacosamide-glucuronide, isolariciresinol sulfate, S-adenosyl-methionine, and O-desmethyltramadol glucuronide (ΔG_bind = −5.63 to −5.75 kcal·mol^−1^). These interactions were fundamentally characterized by intricate networks of hydrogen bonds with residues including Asp164, Gly163, Gln269, and Tyr264/268. These complexes were frequently reinforced by electrostatic stabilization through interactions with Lys157 and Arg166. Recent medicinal and chemistry-based research [30,31] demonstrates the convergence of structurally heterogeneous ligands upon the same S3/S4 pocket, and the BL2 loop (notably Tyr268-Gln269) validates its significance as a primary focus of PL^pro^’s non-covalent inhibition. Furthermore, molecular dynamics simulations substantiated that ligand binding complexes reduced RMDS and increased PL^pro^’s structural rigidity. This trend can be compared to observations for the reference inhibitor GRL0617 and recently for orally bioavailable PL^pro^ inhibitors [31,32,33]. Structural similarity underscores the potential of catechin-7-glucoside as a natural-based high-priority lead. We suggest that it functions through the same conformational stabilization of the BL2 loop as established synthetic inhibitors. Our computational findings corroborate increasing evidence (suggesting) that flavonoid glycosides are capable of forming robust stability and advantageous complexes with SARS-CoV-2 enzymes in silico. However, both the glycosylation architecture and other substituents impact the solvation effects and specific binding interactions [31,32]. Moreover, the potent inhibitory efficacy demonstrated by the crude extract of *A. calamus* and *L. javanica* suggests the potential of synergistic or additive interactions of their diverse phytochemical constituents. In accordance with research on polyphenol-rich botanical formulations, subsequent therapeutic interactions with the host immunological targets and viruses are usually enhanced by the integrated presence of flavonoids, glycosides, and phenolic acids [25,27,34]. Notably, the capacity of catechin-7-glucoside and related metabolites to inhibit activities of both protease and deubiquitinase is significant in highlighting the dual-functionality of PL^pro^ inhibitors that remain as rare and therapeutically relevant agents. Mechanistically, this dual inhibitory activity is important because PL^pro^ acts as an agent that cleaves the viral polyprotein and removes ubiquitin and ISG15 from the immune proteins of the host, therefore, inhibiting interferon responses [11,16]. The simultaneous blockade of these dual functions could stimulate viral replication while effectively restoring innate immune signaling. This profile is currently only featured in a select few established inhibitors, such as certain baicalin derivatives and GRL0617 [33].

The phytochemical profile and historical therapeutic applications of the investigated plants further substantiate the relevance of these discoveries. *L. javanica* is widely recognized in African traditional medicine, and it has been utilized to treat fever, colds, respiratory infections, and inflammation due to its antiviral and immunomodulatory properties [12]. Phytochemical characterizations of *L. javanica* exhibit a rich diversity of flavonoids, iridoid glycosides, phenolic acids, and terpenoids. Several of these compounds have been validated to exert antiviral or anti-inflammatory effects against infections affecting the respiratory system [13,25]. Research conducted prior showed that *L. javanica* has metabolite families capable of modulating the oxidative stress pathways that drive viral pathogenesis and can interact with viral proteases; these findings coincide with our GNPS molecular networking data, which confirmed the prevalence of quercetin and catechin glucuronide clusters [35,36]. Conversely, *A. calamus*, which has been historically utilized in Asian and African traditional medicine for the management of respiratory and inflammatory ailments, is distinguished by its rich content of phenylpropanoids, lignans, and terpenoids, and has a broad spectrum of antimicrobial and antiviral properties [37]. The inhibitory potencies in the current studies were less pronounced than those of catechin-7-glucoside; however, the identification of isolariciresinol sulfate and lacosamide-glucuronide within key molecular families is consistent with earlier studies reporting on the bioactivity of *A. calamus* metabolites against viral targets [38,39]. Additionally, the in situ histochemical evidence of intense ferric chloride-positive staining observed in the leaves of *L. javanica* and the roots of *A. calamus* provides anatomical confirmation of phenolic-based molecular families identified by GNPS networking, with a focus on clusters enriched in derivatives of flavonoid glucuronides, catechins, and phenylpropanoids. Toluidine Blue O metachromasia and phloroglucinol-HCl staining identified lignified tissues and tissues rich in polysaccharides in the same anatomical zones, indicating ferric chloride positivity. This demonstrates that PL^pro^-active metabolites are sequestered within specific phenylpropanoid-linked structural frameworks rather than existing as diffuse cellular pools. The integration of high-resolution metabolomics, GNPS molecular networking, and in situ histochemical data mitigates the uncertainties associated with the detection of stochastic metabolites and validates the reproducibility of phenolic-rich extracts as robust and scientifically sound starting points for the development of antiviral lead compounds. Significantly, the viability of HEK293T cells remained unaffected by both plant extracts at concentrations that effectively inhibited PL^pro^, indicating a favorable safety profile consistent with the plants’ established status in ethnopharmacological practice.

Despite these promising results, several constraints warrant consideration. While the enzymatic assays confirmed the inhibition of PL^pro^ in vitro, the study did not employ whole-virus or complex cellular antiviral models, and therefore, the direct impact on viral replication kinetics and host immunomodulatory signaling remains to be identified. Furthermore, common pharmacological hurdles for polyphenols, such as quercetin glucuronides and catechins, include low bioavailability, rapid metabolism, and restricted membrane penetration. To enhance their drug-likeness and clinical viability, future investigations will need to necessitate pharmacokinetic assessment and structural modification, which will likely involve the exploration of deglycosylation, enhanced lipophilicity, or implementing prodrug methodologies [39]. Recent advancements in orally bioavailable PL^pro^ inhibitors that stabilize the BL2 loop create a strategic blueprint for improving flavonoid-based scaffolds [30].

Furthermore, the activity seen with the whole-plant extracts points to potential synergistic effects across elements, highlighting the importance of additional research incorporating combination testing, bioassay-guided fractionation, and systems-level antiviral evaluation.

## 5. Conclusions

This integrated investigation establishes that *L. javanica* and *A. calamus* form part of the hallmarks of African traditional medicine due to their specialized bioactive metabolites capable of potent inhibition of SARS-CoV-2 papain-like protease (PL^pro^). Through the utilization of a robust, multi-disciplinary pipeline encompassing untargeted metabolomics, GNPS molecular networking, molecular docking, high-resolution 200 ns molecular dynamics simulations, MM-GBSA energy calculations, and in vitro dual-activity assays, the emergent leading natural inhibitor was catechin-7-glucoside. The compound demonstrated potent dose-dependent inhibitory activity against PL^pro^’s protease and deubiquitinase functions, characterized by stable and favorable binding interactions. Its mechanism of action involves the stabilization of the BL2 loop, which mirrors the inhibitory strategy of the reference inhibitor GRL0617. This structural homology further substantiates its potential as a non-covalent PL^pro^ inhibitor. Significantly, both plant crude extracts displayed clear inhibitory activity while exhibiting a total absence of detectable cytotoxicity. Importantly, in situ histochemical profiling provided spatial and developmental validation of the metabolomic data. These findings demonstrate that the phenolic- and flavonoid-rich molecular families identified through GNPS are anatomically sequestered within lignified and phenylpropanoid-active tissues. The convergence of high-resolution chemical profiling, anatomical localization, and functional inhibition significantly boosts the biological relevance and reproducibility of the plant-derived catechin- and phenylpropanoid-based scaffolds as targeted antiviral leads for PL^pro^. The alignment of our data with traditional respiratory treatments underscores the potential of *L. javanica* and *A. calamus* as safe and accessible antiviral bioactive compounds. Collectively, these findings provide a compelling rationale for exploring catechin-derived polyphenols and associated phytochemicals as points of reference for developing novel PL^pro^-targeted therapeutics. Subsequent investigations encompassing validation of antiviral cell-based models, comprehensive pharmacokinetic profiling, and medicinal chemistry optimization will be imperative to translate these natural leads into clinically viable antiviral agents.

## Figures and Tables

**Table 1:** Top 3 significant metabolites (VIP, Log_2_(FC), and −log_10_q-value) identified in *A. calamus* and *L. javanica* extracts (ESI^+^ mode; ESI^−^ mode), ranked by affinity/(ΔGbind/kcal/mol) score.

Metabolites	HMDB ID	Formula	*m*/*z*	RT [min]	Log_2_(FC)	*T*-Test	FDR Log_10_ (p-adj)	VIP	Affinity Score (ΔGbind/kcal/mol)
ESI^+^ mode									
O-Desmethyltramadol glucuronide	HMDB0060856	C_21_H_31_NO_8_	448.196	3.259	−6.194	2.123 × 10^−5^	4.673	1.758	−5.632
Catechin 7-glucoside	HMDB0037949	C_21_H_24_O_11_	453.138	3.177	−5.356	3.285 × 10^−3^	2.484	1.591	−6.427
Quercetin 3-O-glucuronide	HMDB0029212	C_21_H_18_O_13_	479.081	4.241	9.420	6.675 × 10^−8^	7.176	2.186	−5.698
ESI^−^ mode									
S-Adenosyl-methionine	CSID31982	C_15_H_22_N_6_O_5_S	433.099	3.365	9.506	3.434 × 10^−5^	4.464	1.741	−5.751
Isolariciresinol sulfate	HMDB0240703	C_20_H_24_O_9_S	439.101	3.785	7.935	7.094 × 10^−5^	4.149	1.588	−5.659
Lacosamide-glucuronide	HMDB0060829	C_18_H_24_N_2_O_10_	463.109	3.594	7.853	8.637 × 10^−6^	5.064	1.604	−5.711

Key: ΔGbind values denote the predicted binding free energy (kcal/mol) from molecular docking. More negative values indicate stronger and more favorable ligand–protein interactions. VIP = Variable Importance in Projection; Log_2_(FC) = log_2_ fold change; −log_10_q-value = false discovery rate–adjusted significance level.

## Data Availability

The original data is included in the article; further inquiries can be directed to the corresponding author.

## Supplementary Materials

The following supporting information can be downloaded at: https://www.mdpi.com/article/10.3390/life16030373/s1, Figure S1: Representative base peak intensity (BPI) chromatograms obtained from *A. calamus* and *L. javanica* extracts acquired by UPLC-MS/MS in positive (ESI^+^) and negative (ESI^−^) electrospray ionization modes. The corresponding GNPS job links for the mzML datasets generated from these UPLC-MS/MS analyses (ESI^+^ and ESI^−^ modes) are presented for transparency and reproducibility.

## Abbreviations

The following abbreviations are used in this manuscript:

ANOVA	Analysis of Variance
BL2 loop	Blocking Loop 2
CI	Cell Index
CPPTRAJ	AMBER trajectory analysis module
CSV	Comma-Separated Values
DMEM	Dulbecco’s Modified Eagle’s Medium
DMSO	Dimethyl Sulfoxide
DTT	Dithiothreitol
ESI^+^	Positive Electrospray Ionization
ESI^−^	Negative Electrospray Ionization
FBMN	Feature-Based Molecular Networking
FBS	Fetal Bovine Serum
FDR	False Discovery Rate
GAFF2	General AMBER Force Field 2
GNPS	Global Natural Products Social
HMDB	Human Metabolome Database
IC_50_	Half-Maximal Inhibitory Concentration
LC-MS/MS	Liquid Chromatography–Tandem Mass Spectrometry
MM-GBSA	Molecular Mechanics Generalized Born Surface Area
*m*/*z*	Mass-to-Charge Ratio
NPT	Isothermal–Isobaric Ensemble
NVT	Canonical Ensemble
PDB	Protein Data Bank
PL^pro^	Papain-Like Protease
PME	Particle Mesh Ewald
RMSD	Root-Mean-Square Deviation
RT	Retention Time
RTCA	Real-Time Cell Analyzer
SD	Standard Deviation
TIP3P	Transferable Intermolecular Potential 3-Point Water Model
UPLC-MS/MS	Ultra-Performance Liquid Chromatography–Tandem Mass Spectrometry
VIP	Variable Importance in Projection

## References

[1] Wishart D.S., Feunang Y.D., Marcu A., Guo A.C., Liang K., Vazquez-Fresno R., Sajed T., Johnson D., Li C., Karu N. (2018). HMDB 4.0: The Human Metabolome Database for 2018. Nucleic Acids Res..

[2] Hanwell M.D., Curtis D.E., Lonie D.C., Vandermeersch T., Zurek E., Hutchison G.R. (2012). Avogadro: An advanced semantic chemical editor, visualization, and analysis platform. J. Cheminform..

[3] Berman H.M., Westbrook J., Feng Z., Gilliland G., Bhat T.N., Weissig H., Shindyalov I.N., Bourne P.E. (2000). The Protein Data Bank. Nucleic Acids Res..

[4] Trott O., Olson A.J. (2010). AutoDock Vina: Improving the speed and accuracy of docking with a new scoring function, efficient optimization, and multithreading. J. Comput. Chem..

[5] Fu Z., Huang B., Tang J., Liu S., Liu M., Ye Y., Liu Z., Xiong Y., Zhu W., Cao D. (2021). The complex structure of GRL0617 and SARS-CoV-2 PLpro reveals a hot spot for antiviral drug discovery. Nat. Commun..

[6] Tian C., Kasavajhala K., Belfon K.A.A., Raguette L., Huang H., Migues A.N., Bickel J., Wang Y., Pincay J., Wu Q. (2020). ff19SB: Amino-Acid-Specific Protein Backbone Parameters Trained against Quantum Mechanics Energy Surfaces in Solution. J. Chem. Theory Comput..

[7] Narayanan A., Sellers S.A., Jacobson K., Burkard C., Kindrachuk J., Safronetz D., Brueggemann A.B., van Doremalen N., Falzarano D. (2022). Identification of SARS-CoV-2 inhibitors targeting Mpro and PLpro using a cell-based assay. Commun. Biol..

[8] Mani J.S., Johnson J.B., Steel J.C., Broszczak D.A., Neilsen P.M., Walsh K.B., Naiker M. (2020). Natural product-derived phytochemicals as potential agents against coronaviruses: A review. Virus Res..

[9] Luo H., Tang Q.L., Shang Y.X., Liang S.B., Yang M., Robinson N., Liu J.P. (2020). Can Chinese Medicine Be Used for Prevention of COVID-19? A Review of Historical Classics, Research Evidence and Current Prevention Programs. Chin. J. Integr. Med..

[10] Low Z.S., Yip Y.S., Wong C.C., Seow W.L., Ng H.M., Tan K.S., Tan L.W.L., Tan Y., Goh S.S., Cheong W.F. (2023). COVID-19 Therapeutic Potential of Natural Products. Int. J. Mol. Sci..

[11] Rut W., Groborz K., Zhang L., Sun X., Zmudzinski M., Pawlik B., Wang X., Jochmans D., Neyts J., Młynarski W. (2020). Activity profiling and crystal structures of inhibitor-bound SARS-CoV-2 papain-like protease: A framework for anti-COVID-19 drug design. Sci. Adv..

[12] Almeida M.C., Pina E.S., Hernandes C., Zingaretti S.M., Taleb-Contini S.H., Salimena F.R.G., Slavov S.N., Haddad S.K., França S.C., Pereira A.M.S. (2018). Genetic diversity and chemical variability of *Lippia* spp. (Verbenaceae). BMC Res. Notes.

[13] Rajput S.B., Tonge M.B., Karuppayil S.M. (2014). An overview on traditional uses and pharmacological profile of *Acorus calamus* Linn. (Sweet flag) and other *Acorus* species. Phytomedicine.

[14] Makhafola M.A., Naidoo C.M., Obi C.L., Iweriedor B.C., Olaokun O.O., Prinsloo E., Zubair M.S., Mkolo N.M. (2026). Identifying Key Metabolites in South African Medicinal Plants Using Dual Electrospray Ionization Metabolomics. Plants.

[15] Shin D., Mukherjee R., Grewe D., Bojkova D., Baek K., Bhattacharya A., Schulz L., Widera M., Mehdipour A.R., Tascher G. (2020). Papain-like protease regulates SARS-CoV-2 viral spread and innate immunity. Nature.

[16] Genheden S., Ryde U. (2015). The MM/PBSA and MM/GBSA methods to estimate ligand-binding affinities. Expert Opin. Drug Discov..

[17] Roe D.R., Cheatham T.E. (2013). PTRAJ and CPPTRAJ: Software for processing and analysis of molecular dynamics trajectory data. J. Chem. Theory Comput..

[18] Morris G.M., Huey R., Olson A.J. (2008). Using AutoDock for ligand-receptor docking. Curr. Protoc. Bioinform..

[19] Ma C., Sacco M.D., Xia Z., Lambrinidis G., Townsend J.A., Hu Y., Meng X., Szeto T., Ba M., Zhang X. (2021). Discovery of SARS-CoV-2 Papain-like Protease Inhibitors through a Combination of High-Throughput Screening and a FlipGFP-Based Reporter Assay. ACS Cent. Sci..

[20] Case D.A., Aktulga H.M., Belfon K., Ben-Shalom I.Y., Brozell S.R., Cerutti D.S., Cheatham T.E., Cisneros G.A., Cruzeiro V.W.D., Darden T.A. (2022). AMBER 2022.

[21] Jorgensen W.L., Chandrasekhar J., Madura J.D., Impey R.W., Klein M.L. (1983). Comparison of simple potential functions for simulating liquid water. J. Chem. Phys..

[22] Wang J., Wolf R.M., Caldwell J.W., Kollman P.A., Case D.A. (2004). Development and testing of a general AMBER force field. J. Comput. Chem..

[23] Darden T., York D., Pedersen L. (1993). Particle mesh Ewald: An *N*·log(*N*) method for Ewald sums in large systems. J. Chem. Phys..

[24] Wolfender J.-L., Marti G., Thomas A., Bertrand S. (2015). Current Approaches and Challenges for the Metabolite Profiling of Complex Natural Extracts. J. Chromatogr. A.

[25] Khazeei Tabari M.A., Iranpanah A., Bahramsoltani R., Rahimi R. (2021). Flavonoids as Promising Antiviral Agents against SARS-CoV-2 Infection: A Mechanistic Review. Molecules.

[26] Yang J.Y., Ma Y.X., Liu Y., Peng X.J., Chen X.Z. (2023). A Comprehensive review of Natural Flavonoids with Anti-SARS-CoV-2 Activity. Molecules.

[27] Dejani N.N., Elshabrawy H.A., Bezerra Filho C.D.S.M., de Sousa D.P. (2021). Anticoronavirus and Immunomodulatory Phenolic Compounds: Opportunities and Pharmacotherapeutic Perspectives. Biomolecules.

[28] Varghese A., Liu J., Liu B., Guo W., Dong F., Patterson T.A., Hong H. (2025). Analysis of Structures of SARS-CoV-2 Papain-like Protease Bound with Ligands Unveils Structural Features for Inhibiting the Enzyme. Molecules.

[29] Cherrak S.A., Merzouk H., Mokhtari-Soulimane N. (2020). Potential bioactive glycosylated flavonoids as SARS-CoV-2 main protease inhibitors: A molecular docking and simulation studies. PLoS ONE.

[30] Lu Y., Yang Q., Ran T., Zhang G., Li W., Zhou P., Tang J., Dai M., Zhong J., Chen H. (2024). Discovery of Orally Bioavailable SARS-CoV-2 Papain-Like Protease Inhibitor as a Potential Treatment for COVID-19. Nat. Commun..

[31] Kumar S., Paul P., Yadav P., Kaul R., Maitra S.S., Jha S.K., Chaari A. (2022). A Multi-Targeted Approach to Identify Potential Flavonoids against Three Targets in the SARS-CoV-2 Life Cycle. Comput. Biol. Med..

[32] Rao P., Patel R., Shukla A., Parmar P., Rawal R.M., Saraf M., Goswami D. (2022). Identifying Structural–Functional Analogue of GRL0617, the Only Well-Established Inhibitor for Papain-Like Protease (PLpro) of SARS-CoV-2 from the Pool of Fungal Metabolites Using Docking and Molecular Dynamics Simulation. Mol. Divers..

[33] Lin C., Tsai F.J., Hsu Y.M., Ho T.J., Wang G.K., Chiu Y.J., Ha H.A., Yang J.S. (2021). Study of Baicalin toward COVID-19 Treatment: In Silico Target Analysis and In Vitro Inhibitory Effects on SARS-CoV-2 Proteases. Biomed. Hub.

[34] Mhatre S., Gurav N., Shah M., Patravale V. (2021). Entry-Inhibitory Role of Catechins against SARS-CoV-2 and Its UK Variant. Comput. Biol. Med..

[35] Suleman Z., Engwa G.A., Shauli M., Musarurwa H.T., Katuruza N.A., Sewani-Rusike C.R. (2022). Neuroprotective Effects of *Lippia javanica* (*Burm.F*.) *Spreng*. Herbal Tea Infusion on Lead-Induced Oxidative Brain Damage in Wistar Rats. BMC Complement. Med. Ther..

[36] Kaul R., Paul P., Kumar S., Büsselberg D., Dwivedi V.D., Chaari A. (2021). Promising Antiviral Activities of Natural Flavonoids against SARS-CoV-2 Targets: Systematic Review. Int. J. Mol. Sci..

[37] Ashrafi S., Rahman M., Ahmed P., Alam S., Hossain M.A. (2022). Prospective Asian plants with corroborated antiviral potentials: Position standing in recent years. Beni-Suef Univ. J. Basic Appl. Sci..

[38] Huang Y., Li Z., Ma Y., Wu Q., Kong J., Zhao L., Li S., Li J. (2024). Screening for Active Compounds of *Acorus calamus* against SARS-CoV-2 Viral Protease and Mechanism Prediction. Pharmaceuticals.

[39] Frenț O.-D., Stefan L., Morgovan C.M., Duteanu N., Dejeu I.L., Marian E., Vicaș L., Manole F. (2024). A Systematic Review: Quercetin—Secondary Metabolite of the Flavonol Class, with Multiple Health Benefits and Low Bioavailability. Int. J. Mol. Sci..

