# Supplementary material for: Metabolites from South African Medicinal Plants as Dual-Function Inhibitors of the SARS-CoV-2 Papain-like Protease (PLpro)
Source: Life (Basel). 2026 Feb 25;16(3):373. doi: 10.3390/life16030373 (PMC13028120; doi:10.3390/life16030373)
Supplement: Supplementary file 1 [file life-16-00373-s001.zip › life-4146183-supplementary.pdf]

Supplementary material

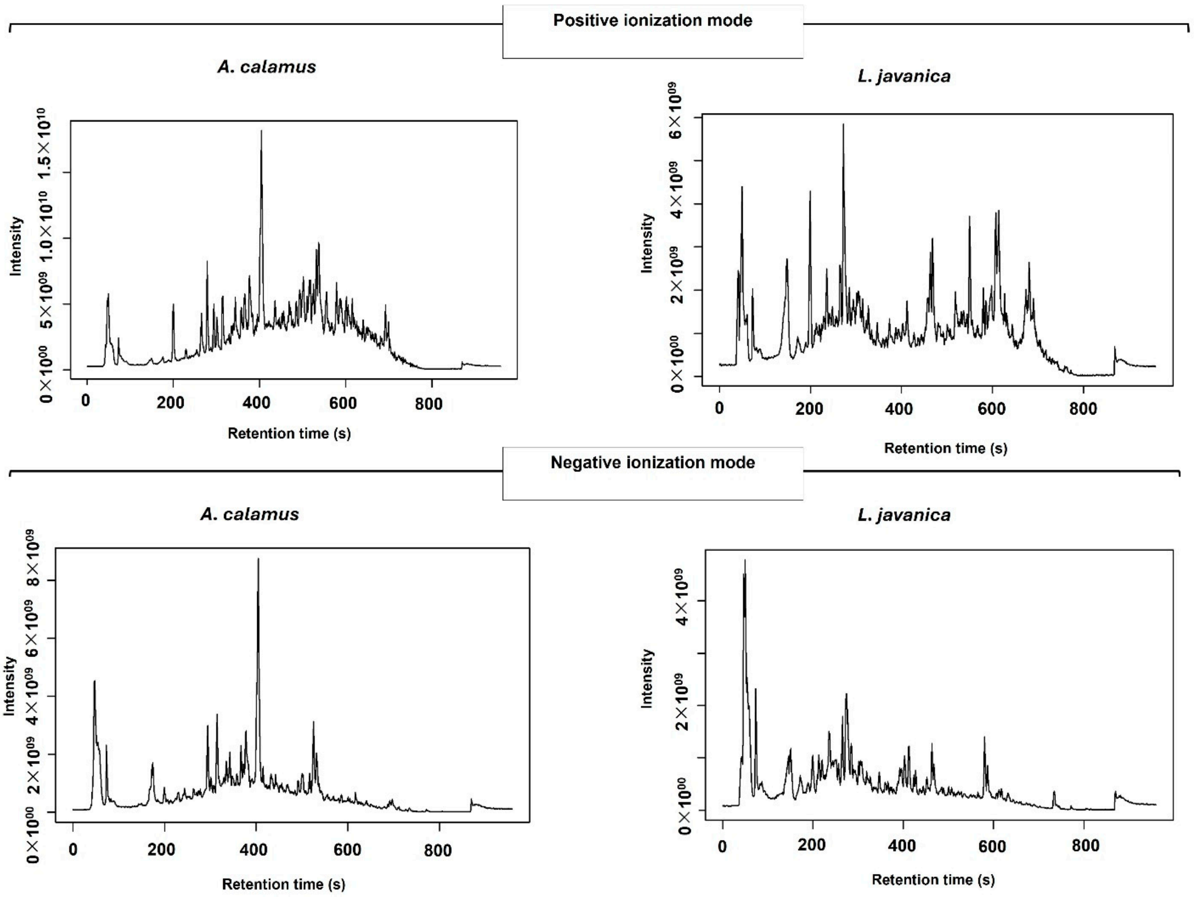

Figure S1: Representative base peak intensity (BPI) chromatograms obtained from *A. calamus* and *L. javanica* extracts acquired by UPLC-MS/MS in positive (ESI^+) and negative (ESI^-) electrospray ionization modes. The corresponding GNPS job links for the mzML datasets generated from these UPLC-MS/MS analyses (ESI^+ and ESI^- modes) are presented for transparency and reproducibility.

GNPS datasets:

Here is the link to the row data:

https://drive.google.com/drive/folders/1_ktuDUNk0pGI-ZVdbEi8yQ2AKaPsd1vF?usp=sharing
